# Phase I and registry study of autologous bone marrow concentrate evaluated in PDE5 inhibitor refractory erectile dysfunction

**DOI:** 10.1186/s12967-019-02195-w

**Published:** 2020-01-14

**Authors:** Mark Bieri, Elias Said, Gabrielle Antonini, Donald Dickerson, Jorge Tuma, Courtney E. Bartlett, Amit N. Patel, Alexander Gershman

**Affiliations:** 1 Regenerative Health LLC, Las Cruces, NM USA; 2 Anonini Urology, Rome, Italy; 3 Creative Medical Health, San Diego, CA USA; 4 Monterrico Clinic & San Felipe Clinic, Lima, Peru; 5 University of Arizona, Tucson, AZ USA; 6 University of Utah - Bioengineering, Salt Lake City, UT USA; 7 Institute for Advanced Urology LLC/University of California Los Angeles, Los Angeles, CA USA

**Keywords:** Bone, Marrow, Erectile, Dysfunction

## Abstract

**Background:**

Bone marrow mononuclear cells have been successfully utilized for numerous regenerative purposes. In the current study, patients suffering from erectile dysfunction (ED) unresponsive to phosphodiesterase 5 inhibitors were administered autologous bone marrow concentrate delivered intracavernously utilizing a point of care FDA cleared medical device.

**Methods:**

A total of 40 patients were treated in the primary trial and 100 in the clinical registry, with the longest follow up of 12 months.

**Results:**

Minimal treatment associated adverse effects where observed related to short term bruising at the site of harvest or injection. No long-term adverse events were noted related to the intervention. Mean improvements in IIEF-5 score were 2 in the Caverstem 1.0 low dose group, 3 in the high dose Caverstem 1.0 group and 9 in the Caverstem 2.0 group. Furthermore, improvements peaked by 3 months and maintained at 6 months follow-up.

**Conclusion:**

These data support the safety and efficacy of point of care, minimally to non-manipulated, non-expanded bone marrow concentrate for the treatment of ED.

*Trial registration* Funded by Creative Medical Health, Inc.; Clinicaltrials.gov number: NCT03699943; https://clinicaltrials.gov/ct2/show/NCT03699943?term=caverstem&rank=1; initially registered December 12, 2015.

## Background

Cardinal features of erectile dysfunction (ED) are inability to achieve and keep an erection sufficient to perform the sexual act. It is widely accepted that ED affects physical and psychological health and has a significant impact on the life quality of sufferers and their partners [1–5]. It is recognized that ED is one of the major complications of chronic inflammatory conditions such as type 2 diabetes [6–8], as well as atherosclerotic disease [9]. The pathogenesis of diabetic ED is multifactorial and complicated, involving persistent damage to the vascular endothelium and smooth muscle and cavernous fibrosis [10].

Currently, oral phosphodiesterase-5 inhibitors (PDE5i) are the initial treatment for patients with ED. Unfortunately, about 35% of patients do not respond. In these patients, diabetes and the associated inflammation, is one of the most common causes of failure to respond to PDE5i [11]. Other reasons for unresponsiveness to PDE5 inhibitors that have been put forth include the presence of restricted inflow of blood into the cavernosa usually as a result of atherosclerosis of the iliac-pudendal—cavernosal arterial vessels, or nerve damage in which nitric oxide is not produced, or smooth muscle atrophy where the remaining corporal tissue is insufficient to allow tumescence to occur [12]. Patients which are refractory to PDE5i, resort to other treatments which are increasingly invasive and include vacuum pumps, penile prostheses, and intracavernosal injections with vasodilators, and vascular surgery. Vacuum pumps may be difficult for some men to use, do not allow for spontaneous, natural erections to occur, and may cause penile trauma if used improperly. Implantation of penile prostheses is invasive, expensive, and irreversible and can cause penile deformity. Intracavernosal injections of vasoactive drugs are satisfactory or effective in 30 to 90 percent of men, but they can be associated with pain, priapism, penile hematomas, and fibrosis. Clinical interest in penile revascularization surgery stems from the widely reported link between ED and atherosclerotic vascular disease [13, 14]. Unfortunately, the success rate of vascular surgery has been reported to be highly variable and has raised questions concerning the appropriate means for diagnosis of arteriogenic ED, and the safety and feasibility of stent-based therapies [15]. Since the second- and third-line ED treatments all involve invasive or cumbersome surgeries and devices, cell-based therapy is uniquely positioned to address the unmet medical need for alternative ED therapies that might allow for restoration of natural erectile function.

Cell-based therapies for treating ED, including bone marrow cells as well as other tissue sources have been reported. Kendirci et al. used bone marrow cells that were isolated on the basis of expression of the p75 nerve growth factor receptor using magnetic activated cell sorting [16]. They chose this population based on possible enhancement of neurogenic potential. Intra-cavernous administration of these cells into a rat bilateral cavernous nerve crush injury model was performed. At 4-week follow-up, improvement in erectile function in response to the stem cell treatment was observed on the basis of changes in mean intra-cavernous-to-mean arterial pressure ratio and total intra-cavernous pressure. Therefore, non-expanded bone marrow cells afforded significant repair of erectile function in animals.

Using mesenchymal stem cells (MSC) in the treatment of ED is shown to have therapeutic benefits not only because these cells are known to secrete various growth factors that are beneficial in ED such as IGF-1 [17–19], VEGF [20], and FGF-2 [21], but also because of their anti-inflammatory activities [22], as well as possibility of differentiating into tissue relevant to the penile architecture [23]. To assess whether bone marrow derived MSC had a therapeutic effect on diabetes-induced ED, Qiu et al. performed intra-cavernous administration of these cells. Four weeks after administration, the ratio of intra-cavernous pressure and mean arterial pressure, as well as smooth muscle and endothelial cell compartment was significantly upregulated compared to controls. Cell tracking experiments revealed that the MSC were retained for at least 4 weeks post-injection and showed expression of endothelial and smooth muscle cell markers, suggesting that the stem cells can differentiate [24].

Circulating endothelial progenitor cells (EPC) are a subset of bone marrow progenitor cells with the capacity to promote blood vessel repair by differentiating into endothelial cells. Significantly, EPC numbers are significantly lowered in circulation of patients with erectile dysfunction, and a significant correlation was found between scores on the International Index of Erectile Function questionnaire and circulating EPC levels [25]. To test their efficacy in an animal model, EPC were injected intracavernously and were found to migrate to the arteries, to preserve smooth muscle function, and restored erectile function [26]. In another study, CD133 + progenitor cells isolated from bone marrow were found to afford histological and functional recovery in an animal model of cavernous nerve injury [27]. Altogether, these findings elucidate the therapeutic potential of various populations of bone marrow-derived cells for treating ED.

The current study reports initial clinical investigations evaluating safety and signals of efficacy of autologous bone marrow concentrate administered to patients with PDE5i refractory ED.

## Methods

### Study goals and objective

*The goal of this clinical trial is to evaluate the injection of autologous bone marrow concentrate (Caverstem 1.0*—*low dose versus high dose) into patients with refractory PDE5i ED of vascular origin. The primary endpoint is safety as measured by adverse events monitored by an independent medical monitor and change in* International Index of Erectile Function—(IIEF-5) questionnaire scoring from baseline to 6 months. Secondary endpoints including Doppler ultrasound and dynamic infusion caversometery was evaluated in the clinical trial from baseline up to 6 months [28, 29] in USA. A parallel clinical registry has also been created which includes patients with similar causes of ED that have been injected with bone marrow concentrate (Caverstem 2.0) and have been followed for same time period as the primary end-point of the study and will be included in the safety and data analysis enrolled globally.

### Patients

Men age 18 years or older that have been diagnosed with erectile dysfunction. Eligibility of the patient is finalized based on the physician’s recommendation after the Visit 1 evaluation. The diagnosis of ED and suitability for this procedure is based on physical examination, medical history, including sexual history, laboratory assessment, International Index of Erectile Function—(IIEF-5) questionnaire scoring (severe (5–7), moderate (8–11), mild to moderate (12–16), and mild (17–21)), nocturnal penile tumescence testing. The diagnosis of vascular ED was based on physical examination (including heart rate, EKG, and blood pressure monitoring) and medical history, including sexual history, laboratory assessment, IIEF questionnaire scoring, nocturnal penile tumescence, Doppler ultrasonography, and dynamic infusion cavernosonometry. Other patient criteria for inclusion was: chronic organic ED duration at least 0.5 years, diagnosis of ED based on Doppler ultrasound and/or dynamic infusion cavernosonometry, baseline (IIEF-5) score of < 21, oral medications and intracavernous pharmacological approaches have been deemed ineffective, contraindicated or cannot be tolerated, concurrently undergoing treatment with testosterone. Exclusion criteria consisted of: subjects using any new medications/drugs with known effects on erectile function within 4 weeks of the study period, including certain antidepressants, antihistamines, diuretics, and beta-blockers, subjects using herbal remedies for addressing erectile dysfunction within 1 month of study initiation, subjects with penile prosthesis or other urinary prosthesis, subjects with penile anatomical deformities (e.g. Peyronie’s disease) or history of priapism, previous penile surgeries for erectile dysfunction, premature ejaculation or penile enlargement, diagnosis of psychogenic ED as determined by nocturnal tumenscence testing, presenting with uncontrolled or severe disease, including cardiovascular disease, diabetes, liver disease, uncontrolled hypertension or hypotension (systolic blood pressure > 170 or < 90 mm Hg, and diastolic blood pressure > 100 or < 50 mm Hg), suffered a cardiovascular event within 6 months prior to study initiation, current or previous malignancy other than non-melanoma skin cancer (successfully treated or treatable by curative excision or other local curative therapy), diagnosis of a systemic autoimmune disorder, receiving immunosuppressant medications. Institutional Review Approval Los Angeles Biomedical Research Institute at Harbor UCLA Medical Center# 21511-01 and 21760-01. Independent Medical Monitor: Peter Liu MD. Statistical analysis of continuous variables was performed using paired t-tests and one way ANOVA was performed using GraphPad Software (San Diego, CA).

### Bone marrow aspiration and concentration—Caverstem 1.0—clinical trial

After informed consent was obtained the patient was administered hydrocodone 10 mg (for analgesia) and 0.5 mg alprazolam (for anxiolysis) orally 30 min prior to the procedure. Patients were placed in a lateral decubitus/prone position. Sterile preparation and draping was performed. Lidocaine 1% up to 10 cc was injected into the epidermis down into the periosteum. A Jamshidi-type needle was used for bone marrow aspiration. Bone marrow was aspirated into a 10 cc syringe that was pre-loaded with 3–4 mL of anti-coagulant. To aspirate more marrow, the needle was rotated 45° to reorient the bevel. After a full rotation of the needle at this level, the needle can be withdrawn approximately 1 cm toward the surface for further aspiration. In some situations, several perforations can be made through the same skin opening, approximately 2 cm apart. Once bone marrow procurement was complete, direct pressure with sterile gauze is applied to prevent bleeding and a small sterile bandage was applied over the needle entry site. The target volume of bone marrow aspirate is 30 mL-low dose group or 60 mL-high dose group per patient. It was estimated that approximately 3–5 aspirations will be needed to obtain sufficient bone marrow. The Magellan^®^ device was used for concentrating stem cells from bone marrow aspirate according to the manufacturer’s instructions. The Magellan^®^ device is a fully automated and closed system comprising a microprocessor-controlled centrifuge and syringe pumps that concentrate specific cellular populations. Bone marrow is dispensed into centrifuge chambers for rapid and automatic enrichment of cellular fractions to yield a bedside prepared product rich in platelets, hematopoietic stem cells (HSC) and mesenchymal stem cells (MSC) in as little as 15 min. The Magellan^®^ device yields a 3–10 cc injectable unit of platelet and stem cell enriched plasma for the physician’s use for intracorporal injection into the same patient. According to a published report where bone marrow was enriched with the Magellan^®^ 27 mL of bone marrow yielded approximately 1.7 × 10^8^ cells concentrated into 3 mL [30]. In the current study, the Magellan^®^ device was utilized to concentrate a dose of up to 10^8^ cells (post-enrichment) into 3 mL-low dose group or 6 mL-high dose group for intracavernosal injection from a starting (pre-enrichment) volume of 30 or 60 mL bone marrow. This procedure was conducted in the examination room at the patient’s bedside.

### Intracavernosal injection—Caverstem 1.0—clinical trial

Following extraction of 3 mL-low dose group or 6 mL-high dose group of sterile bone marrow concentrate using the Magellan device, two 1/2-in. 25-gauge needles were filled with 1.5 or 3 mL of the bone marrow concentrate. The injection site was cleansed with an alcohol swab prior to injection. The cells were injected into both corpora cavernosum along the dorso-lateral aspect of the proximal third of the penis. Care was taken to avoid any area where there are visible veins. After the injections, sterile gauze was placed with pressure on the injection site to prevent bleeding. The injection sites were inspected, and hemostasis was confirmed.

### Bone marrow aspiration and concentration—Caverstem 2.0—clinical registry

After informed consent was obtained and the patient was brought to the procedure room, placed in a supine position, and was sterilely prepped and draped. A 1% lidocaine penile nerve block was placed. Patient was then placed in prone position. Lidocaine 1% up to 10 cc was injected into the epidermis down into the periosteum at the level of the posterior iliac crest. The Marrow Cellutions kit was heparinized using unfractionated heparin solution of 5000 U/mL with a total of 2 cc used including the syringe(s) used for aspirating the bone marrow. An 11-gauge access trocar was introduced through the cortex of the posterior Iliac crest. A 1 cc aspiration was done to ensure access into marrow space. To promote safety, the blunt stylet was used to access a distal point in the ileum. The black top closed aspiration cannula was then placed and secured tightly to prevent any air leak. The external leverage housing system was unwound until contact was made with the skin. This allowed leverage for the repositioning of the recovery of stem and progenitor cells from a different level within the marrow with each complete turn of the system. Negative pressure was applied by carefully withdrawing the syringe plunger. Twenty milliliters of bone marrow aspirate was obtained. Only 1–2 cc of marrow is aspirated at each position in the ileum, turning the blue T handle counterclockwise 360° after each 1–2 cc aspiration. This exposed the aspiration cannula tip to a different marrow area in order to maximize the quality of the bone marrow collection. The black top aspiration cannula was then removed, and the blunt stylet was introduced into the access trochar and secured in locked position. A series of clockwise and counterclockwise rotations were used as the trochar was gently withdrawn. Hemostasis was obtained and a dressing applied. The bone marrow aspirate was not manipulated in anyway and was not allowed to leave the sterile field.

### Intracavernosal injection—Caverstem 2.0—clinical registry

The penile shaft was prepped with bacteriocidal solution. An elastic tourniquet was placed under moderate tension at the base of the penis. The 10 mL syringe with aspirate drawn from the first portion of the procedure was brought to the field, and a heparinized 21 gauge needle affixed, which was then used to inject 10 mL of the aspirate into one of the penile corporal bodies, and this then repeated on the contra-lateral corporal body in similar fashion with the second 10 mL of the bone marrow aspirate that had been obtained. The elastic tourniquet was held for up to 15 min allowing increased dwell time and then released. A dressing is applied over the corporal injection sites and gentle pressure was applied to minimize local bruising and bleeding. The injection sites were inspected, and hemostasis was confirmed.

## Results

After institutional review board approval and informed consent, 40 men were successfully enrolled in the clinical trial and 100 in the clinical registry. The baseline demographics are low dose versus high dose versus registry: Mean age: 36 ± 5 years/52 ± 12 years/57 ± 15, Hypertension: 5/15/38, Diabetes-non-insulin: 1/10/29, Hyperlipidemia: 2/5/22, Prior smoker: 2/9/35, IIEF-5: 8/9/9. The procedures were well tolerated and no patients reported significant adverse effects (Table 1). All patients had successful harvest of the bone marrow with adequate product available to be injected. Functional bone marrow concentrate analysis was performed utilizing the CFU-f per ml which was 506 ± 102 for Caverstem 1.0 and 3160 ± 297 for Caverstem 2.0. There were no issues related to injecting the bone marrow into the penis and was tolerated by the patients. The was some pain and bruising at both the harvest site and injection site in all groups. However, this finding did not persist. There was no perception by the patient that the procedure adversely effected their penis. There was no infection or fevers in any of the groups (Table 1). There was no statically significant change in doppler ultrasound and cavernosmetry in the low or high dose group. These measurements were not performed in the clinical registry as part of a standardized operating protocol and hence were not collected or analyzed. There was improvement in the IIEF-5 scoring in all three groups (Table 2).

**Table 1:** Safety—serious and non-serious adverse events up to 6 months post intervention

Caverstem intervention	1.0 low dose	1.0 high dose	2.0
Number of patients	20	20	100
Serious adverse event	0 (0)	0 (0)	0 (0)
Loss of injection product (n)	0 (0)	0 (0)	0 (0)
Total volume injected (mL)	3	6	20
Injection of allocated amount of product (n)	20 (100)	20 (100)	100 (100)
Adverse event	n(%)	n(%)	n(%)
Pain at harvest site^a^	5 (25)	7 (35)	9 (9)
Pain at injection site^a^	6 (30)	6 (30)	5 (5)
Brushing at harvest site^a^	5 (25)	5 (25)	6 (6)
Bruising at injection site^a^	3 (15)	4 (20)	4 (4)
Negative impact on penis^b^	0 (0)	0 (0)	0 (0)
Fevers	0 (0)	0 (0)	0 (0)
Infection	0 (0)	0 (0)	0 (0)

^a^Greater than 24 h post procedure

^b^As perceived by the patient

**Table 2:** Mean international index of erectile function—(IIEF-5) questionnaire

Caverstem intervention	Baseline (range)*	3 month (range)*	6 month (range)*
1.0 low dose	8 (5–16)	11 (5–25)	10 (5–22)
1.0 high dose	9 (5–16)	12 (5–19)	12 (5–20)
2.0	9 (5–15)	18 (10–24)**	18 (10–23)**

* IIEF-5 ED scoring (severe (5–7), moderate (8–11), mild to moderate (12–16), and mild (17–21) dysfunction. **p = 0.001

## Discussion

The current findings support the safety and potential efficacy of autologous, non-expanded bone marrow concentrate for treatment of ED patients. The current study was limited by the heterogeneity of the patient population as to causes of ED even though the primary recruitment was related to vasculogenic ED. Other studies utilizing autologous bone marrow have been confined to patients post prostatectomy. Despite this, we observed significant improvements in IIEF-5 scores which were superior to the isolated bone marrow cells and/or adipose published clinical trials [31, 32]. The mean IIEF-5 scores observed in PDE5i studies have improved on average of by 8.5 compared to 9 which is what we found the in the clinical registry [33]. The use of autologous bone marrow concentrate is enticing not only because of the known secreted growth factors that are beneficial in ED such as IGF-1 [17–19], VEGF [20], and FGF-2 [21], but also because of their anti-inflammatory activities [22], as well as possibility of differentiating into tissue relevant to the penile architecture [23].

Previous clinical studies have supported the safety and potential efficacy of regenerative cell administration into the corpus cavernosum. Yiou et al. studied post-prostatectomy patients administered escalating number of bone marrow mononuclear cells. No serious side effects occurred. At 6 mo versus baseline, significant improvements of intercourse satisfaction and erectile function domains of the International Index of Erectile Function-15 and Erection Hardness Scale (2.6 ± 1.1, 1.3 ± 0.8, p = 0.008) were observed in the total population [34]. In diabetic patients with ED, a study examined 4 patients with refractory ED were included. Two consecutive intracavernous autologous BM-MSC injections were performed. Tolerability was assessed immediately and at 24 h, safety was evaluated for 2 years. The investigators found that procedure was well tolerated and no patients reported significant adverse effects. There was significant improvement of sexual function scores [31].

The use of the Caverstem 1.0 procedure in the clinical trial though safe and somewhat effective may have an unknown impact on the potency of the product as a result of the minimal manipulation. The total amount of bone marrow aspirated in the clinical trial was 30 or 60 mL which was then concentrated into the 3 or 6 mL for injection into the patients compared to the 20 mL in the clinical registry which injected in full into the patient. Despite the volume difference, if only concentration of the cellular product was occurring in the clinical trial then there should not be a difference in the clinical results as seen in the patients compared to what was observed in the Caverstem 2.0 clinical registry. The patients in the clinical registry despite being older appeared to have more benefit. Our team has previously demonstrated that bone marrow concentrate may efficacious in a case report of a ED patient [35]. However, as there are limitations of any open label study using a patient as their own control is the only way to evaluate treatment effect. As all patients in the clinical trial and clinical registry are on optimized medical therapy as determined by the primary care physician and/or urologist before they are consented, the likeliness of a change in post procedure medical therapy having an impact on the patient’s ED is minimal. Also, as all patients in the clinical trial and the clinical registry received a bone marrow aspirate and injection, the placebo effect along with other confounding factors should be similar amongst groups. The data from this clinical registry should continue to be expanded to more sites to verify quality control in performing this procedure and to maintain the utmost patient safety. Further study should be performed to optimize potential responders versus non-responders and duration of positive effect if observed due to the possible need of redosing as progression of other systemic diseases always have an impact on ED.

## Conclusion

The current clinical trial and clinical registry demonstrate the use of autologous bone marrow concentrate in the treatment of PDE5i refractory ED is safe and feasible with promising clinical results.

## Data Availability

Upon request.

## Abbreviations

ED: erectile dysfunction
PDE5i: phosphodiesterase-5 inhibitors
MSC: mesenchymal stem cells
IGF-1: insulin-like growth factor 1
VEGF: vascular endothelial growth factor
FGF-2: fibroblast growth factor 2
EPC: endothelial progenitor cells
IIEF-5: International Index of Erectile Function 5
EKG: electrocardiogram
HSC: hematopoietic stem cells
cGMP: cyclic guanosine monophosphate
eNOS: endothelial nitric oxide
